# Things We Do for No Reason™: Routinely maintaining intravenous access in hospitalized patients

**DOI:** 10.1002/jhm.70191

**Published:** 2025-10-06

**Authors:** Brent Kennis, John P. Gerstenberger, Lara Hayes

**Affiliations:** ^1^ Department of Internal Medicine University of Utah Salt Lake City Utah USA

## Abstract

Peripheral intravenous catheters (PIVCs) are widely used in hospitalized patients and are often maintained even after the need for intravenous therapy has resolved. This article challenges the routine practice of maintaining idle PIVCs in clinically stable patients. While PIVCs offer convenient access for emergent treatment, they are associated with risks—including local infections, phlebitis, and vascular damage—as well as significant patient discomfort. Notably, although PIVCs carry a lower individual risk of bloodstream infection compared with central venous catheters, their widespread use makes them responsible for up to one‐third of *Staphylococcus aureus* catheter‐related bacteremia. Furthermore, idle PIVCs often fail before use, and intraosseous access provides an effective alternative in true emergencies. As many intravenous medications can be safely and effectively administered orally, the continued use of PIVCs in stable patients may offer little benefit while introducing avoidable harms and costs. Clinicians should regularly reassess the need for intravenous access and remove idle PIVCs when appropriate to promote patient safety and comfort.

## CLINICAL SCENARIO

An 80‐year‐old man is admitted to the hospital with viral gastroenteritis and acute kidney injury. A peripheral intravenous catheter (PIVC) is placed for fluid administration. Over the next day, his diarrhea improves, and he is hydrating orally. He remains hospitalized while awaiting disposition. His PIVC is functioning normally with no abnormalities on inspection of the site; however, he finds the PIVC uncomfortable and asks if it can be removed.

## WHY YOU MIGHT THINK ROUTINELY MAINTAINING INTRAVENOUS ACCESS IS NECESSARY

PIVCs are ubiquitous in hospitals worldwide and are a cornerstone of management of various acute medical conditions.^1^ PIVCs are also commonly placed pre‐emptively in case intravenous therapies are needed. Approximately one‐third of PIVCs placed in the emergency department are never used.^2^ Similarly, nearly one quarter of PIVCs in hospitalized patients in North America are maintained but not actively used, the so‐called “idle PIVCs.”^1^ Maintenance of PIVCs in hospitalized patients, even when not used for planned medication administration, offers clinicians convenient and rapid ability to administer intravenous medications in the event of unexpected clinical changes.

## WHY ROUTINELY MAINTAINING INTRAVENOUS ACCESS IS NOT NECESSARY

Despite their ubiquity in the practice of hospital medicine, PIVCs have risk. Most notable is the increased risk of infection. One study reported an incidence rate of 65 episodes per 100,000 catheter‐days for local infection and 4.4 episodes per 100,000 catheter‐days for bloodstream infection deemed secondary to PIVCs.^3^ Although PIVCs are one order of magnitude less likely to cause bacteremia compared with central venous catheters (CVCs) the frequency of their use leaves them responsible for over 1/3rd of catheter‐related *Staphylococcus aureus* bacteremia (SAB) and approximately 20% of all SAB.^4^

Noninfectious complications of PIVCs including pain and phlebitis are common, affecting 10%–20% of patients with PIVCs.^1^, ^5^ Maintaining PIVCs also involves burdensome monitoring and replacement to maintain patency, cause both acute and chronic vascular damage, and can complicate the care of delirious patients.^6^, ^7^ Furthermore, catheter placement and replacement even in the absence of complications can be a source of pain and distress in hospitalized patients.^8^

PIVCs are also less reliable than one would hope if maintained for the purpose of possible emergent therapies. More than one‐third of PIVCs fail before treatment completion and 10% of PIVCs had signs of malfunction in one analysis.^1^, ^3^ Assuming patency in this scenario poses significant risks. Emergent Intraosseous (IO) access can mitigate these risks. In one retrospective study, nearly 20% of inpatients with cardiac arrest at a tertiary hospital were resuscitated with IO access due to the absence or failure of the PIVC.^9^ In multivariable analysis, rates of survival with favorable neurologic status and survival to discharge were not significantly different between groups resuscitated with PIVC compared with IO.^9^ This suggests that maintaining PIVCs in all hospitalized patients in case of rare cardiac arrest provides a false sense of security due to high PIVC failure rate, but also are not necessary due to the efficacy of emergently placed IO access and modern rapid response and vascular access teams. In less emergent changes in clinical status, PIVCs can be placed in hospitalized patients when needed or other routes of administration can be utilized.

Additionally, increasing evidence favors oral routes of administration for medications commonly given intravenously in the hospital (e.g., opioids and antibiotics) for reasons of both cost and efficacy.^10^, ^11^ In these cases, even actively used PIVCs may be appropriate targets for removal if the intravenous medications are more appropriately administered orally.

Lastly, there is a financial burden associated with routinely inserting, maintaining, and monitoring PIVCs as well as managing complications of PIVCs such as infection, though the cost attributable specifically to unnecessary PIVCs is not clear.

## WHEN MAINTAINING INTRAVENOUS ACCESS MIGHT BE HELPFUL

PIVCs remain a safe and life‐saving tool in the management of acutely ill patients in the hospital. Even for patients without an immediate intravenous therapeutic need, intravenous access remains the preferred route of drug administration in the case of cardiac arrest,^12^ and “just in case” PIVCs are appropriate in patients at the highest risk of decompensation as indicated by clinical judgement. Furthermore, maintaining idle PIVCs may be prudent in hospitalized patients with anticipated diagnostic studies requiring intravenous contrast, upcoming procedures requiring sedation, or with uncertain ability to tolerate or absorb oral medications.

## WHAT YOU SHOULD DO INSTEAD

While generally safe, clinicians should appreciate that this routine intervention can cause a variety of complications, including life‐threatening bloodstream infections. When PIVCs are indicated, evidence‐based approaches should be used to reduce the risk of complications, such as clinically indicated replacement.^6^, ^13^ Hospitalists should frequently reassess the indications for established PIVCs and consider removal of idle PIVCs or active PIVCs if the intravenous medications could be more appropriately transitioned to an oral route of administration. The large proportion of patients who remain hospitalized despite medical readiness for discharge is a particularly important group for which harms of PIVCs likely outweigh benefits.^14^ The risks of PIVCs may be under appreciated by clinicians in comparison to other common medical interventions such as urinary catheters and CVCs. Implementation of a validated PIVC assessment tool is one successful strategy to reduce prevalence of idle PIVCs and PIVC complications at a systems level.^15^

## RECOMMENDATIONS

− Place PIVCs only in selected patients who require short‐term intravenous therapies or who are likely to decompensate and require intravenous therapies emergently.
− In hospitalized patients with PIVCs, regularly monitor and reassess the ongoing need for a PIVC.
− Consider PIVC removal in hospitalized patients who are stable, whose medications can be administered orally, and who have no anticipated other need for intravenous access in the immediate future.

## CONCLUSIONS

Returning to the case, the hospitalist should assess the patient's clinical course and response to initial treatment. Given the acute kidney injury on admission, labs should be repeated to understand if the patient is at risk of sudden decompensation due to severe electrolyte disturbances or progressive renal failure requiring additional interventions. The patient's volume status should be assessed to determine whether there is further indication for intravenous fluids. The clinician should also inquire about the patient's ability to tolerate oral hydration and medication administration. If no indication for intravenous therapies is present and the patient is not at high risk for decompensation, the PIVC should be removed to improve patient comfort and reduce the risk of other PIVC‐related complications.

## CONFLICT OF INTEREST STATEMENT

The authors declare no conflicts of interest.
